# Nordihydroguaiaretic acid impairs prostate cancer cell migration and tumor metastasis by suppressing neuropilin 1

**DOI:** 10.18632/oncotarget.13368

**Published:** 2016-11-15

**Authors:** Xin Li, Shengjun Fan, Xueyang Pan, Yilixiati Xiaokaiti, Jianhui Duan, Yundi Shi, Yan Pan, Lu Tie, Xin Wang, Yuhua Li, Xuejun Li

**Affiliations:** ^1^ State Key Laboratory of Natural and Biomimetic Drugs, Department of Pharmacology, School of Basic Medical Sciences, Peking University and Beijing Key Laboratory of Tumor Systems Biology, Peking University, Beijing 100191, China; ^2^ Current address: University of Minnesota, Twin cities, MN 55455, USA

**Keywords:** nordihydroguaiaretic acid, neuropilin 1, cancer cell migration and metastasis, proteomics, prostate cancer

## Abstract

Tumor metastasis is a major cause leading to the deaths of cancer patients. Nordihydroguaiaretic acid (NDGA) is a natural product that has been demonstrated to show therapeutic values in multiple diseases. In this study, we report that NDGA can inhibit cell migration and tumor metastasis via a novel mechanism. NDGA suppresses NRP1 function by downregulating its expression, which leads to attenuated cell motility, cell adhesion to ECM and FAK signaling in cancer cells. Moreover, due to its cross-cell type activity on NRP1 suppression, NDGA also impairs angiogenesis function of endothelial cells and fibronectin assembly by fibroblasts, both of which are critical to promote metastasis. Based on these comprehensive effects, NDGA effectively suppresses tumor metastasis in nude mice model. Our findings reveal a novel mechanism underlying the anti-metastasis function of NDGA and indicate the potential value of NDGA in NRP1 targeting therapy for selected subtypes of cancer.

## INTRODUCTION

Tumor metastasis is a major cause for the deaths of cancer patients [1]. The metastatic cascade is a multistep and complex biological process, dynamically modulated by tumor cells, tumor microenvironment and the interactions between them [2]. For decades, great efforts have been made to understand the mechanisms underlying metastasis and a series of potential therapeutic targets have been identified. However, there are still very few drugs that are developed directly targeting tumor metastasis in clinical therapies [1].

Natural products have been an important source for drug discovery and development for centuries [3]. Nordihydroguaiaretic acid (NDGA), a phenolic compound extracted from creosote bush (*Larrea tridentate*), is proved to have versatile effects on multiple biological processes and potentials in therapeutic applications in a series of diseases [4]. In various cancer models, NDGA has been demonstrated to promote apoptosis and inhibit proliferation by suppressing the activities of lipoxygenase (LOX), insulin-like growth factor 1 receptor (IGF-1R) and human epidermal growth factor receptor 2 (HER2/neu) [5–8]. However, in contrast to the much better understood anti-proliferation activities, the effects of NDGA on cancer cell migration and tumor metastasis were rarely studied. One study reported that NDGA reduces B16 melanoma cell metastasis *in vitro* and *in vivo* [9], but the mechanism underlying this effect remains unclear.

Neuropilin 1 (NRP1) is a single-pass transmembrane protein playing important roles in development, angiogenesis, immunity and cancer [10]. In many types of cancer including breast, prostate, pancreatic, colon and kidney cancer, NRP1 can be found overexpressed and the abnormal expression pattern usually correlates with tumor aggressiveness, metastasis and poor prognosis [11]. It has been demonstrated that NRP1 regulates multiple cellular processes involved in tumor progression, including cell proliferation, migration, invasion, adhesion and even the sensitivity of tumor cells to chemo/radio-therapy, by binding with various cancer-associated growth factors and enhancing activities of respective receptor tyrosine kinases [12–14]. In addition to its co-receptor function mentioned above, recent studies show that NRP1 is able to modulate tumor microenvironment by interacting with integrins and remodeling extracellular matrix (ECM) [15, 16]. In recent years, various approaches targeting NRP1 have been proved to execute anti-tumor effect in both cultured cells and animal models [17–19], indicating NRP1 as a promising drug target in anti-cancer therapy.

In this study, we elucidated the inhibitory effect of NDGA on PC3 cell migration using *in silico*, *in vitro* and *in vivo* studies. We demonstrated that NDGA suppresses NRP1 expression and consequently impairs cell motility and cell adhesion to ECM in cancer cells and attenuates tumor metastasis in nude mice model. Our findings reveal a novel mechanism underlying the anti-metastasis function of NDGA and indicate the potential value of NDGA in NRP1 targeting therapy for selected subtypes of cancer.

## RESULTS

### NDGA inhibits PC3 cell migration

**Figure 1:**
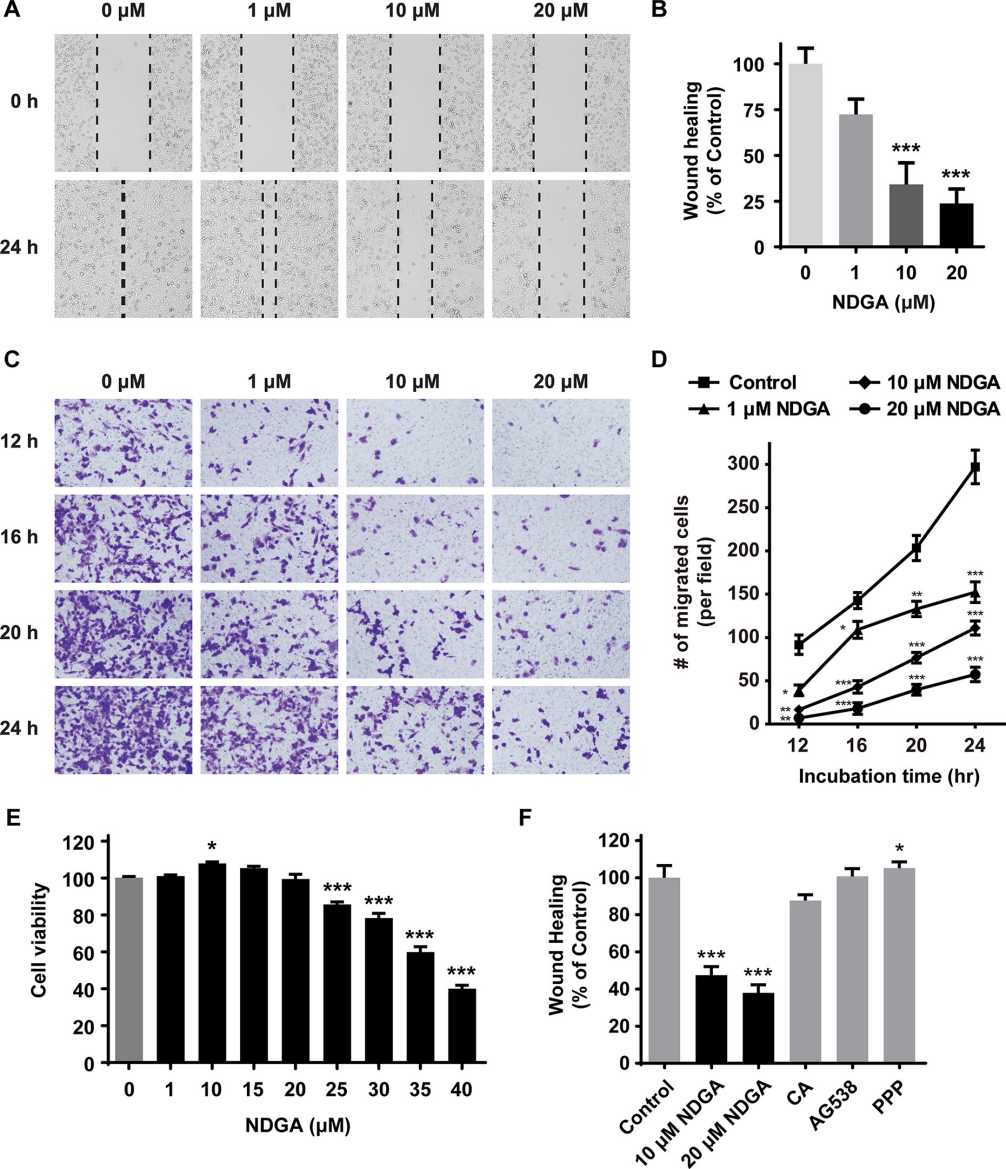
NDGA suppresses cell migration of PC3 cells (**A**) Wound healing assay of PC3 cells treated with different concentrations (0, 1, 10, 20 μM) of NDGA for 24 hours. Representative wound images of each group are shown. (**B**) Quantification of wound healing assay. Migration distance were normalized to control group. Data show mean ± S.E (*n* = 3). ***, *p* < 0.001. (**C**) Transwell assay of cells treated with NDGA for 12, 16, 20 or 24 hours. Representative images of migrated cells of each group are shown. (**D**) Quantification of transwell assay. Data show mean ± S.E (*n* = 3). *p < 0.05, **p < 0.01, ***p < 0.001. (**E**) Cells were treated with indicated doses of NDGA for 24 hours. Cell proliferation was determined using MTS assay. *p < 0.05, ***p < 0.001. (**F**) Wound healing assay was used to measure PC3 cells migration in the presence of NDGA (10 or 20 μM), 30 μM caffeic acid (CA), 4 μM AG538 or 4 μM picropodophyllin (PPP) for 24 hours. Data show mean ± S.E (*n* = 3). *p < 0.05, ***p < 0.001.

Previous studies have shown that NDGA inhibits tumor cell proliferation and induces apoptosis in many cancer models [7, 20]. Here we further investigated the inhibitory effect of NDGA on cell migration in prostate cancer PC3 cells. Exposure to NDGA for 24 hours significantly inhibits PC3 cell migration in a dose-dependent and time-dependent manner (Figure 1A–1D). Moreover, when we measured cell viability after NDGA treatment, we found that NDGA does not attenuate cell proliferation at the concentrations that suppress cell migration (Figure 1E). Previous publications reported that NDGA functions as inhibitor of LOX [21] and IGF-1R [22, 23]. To test whether NDGA attenuates cell motility via these known targets, we introduced some other small molecular inhibitors which could reproduce the known activities of NDGA on LOX or IGF-1R [24] and tested their effects on PC3 cell migration. It turned out that all of these small molecular inhibitors, including LOX inhibitor caffeic acid and IGF-1R inhibitor AG538 and picropodophyllin (PPP), failed to induce suppression on cell migration of PC3 cells (Figure 1F), suggesting that NDGA suppresses cell migration through a novel mechanism other than those known ones.

### Identification of the key proteins contributing to the inhibition of NDGA on cell migration

**Figure 2:**
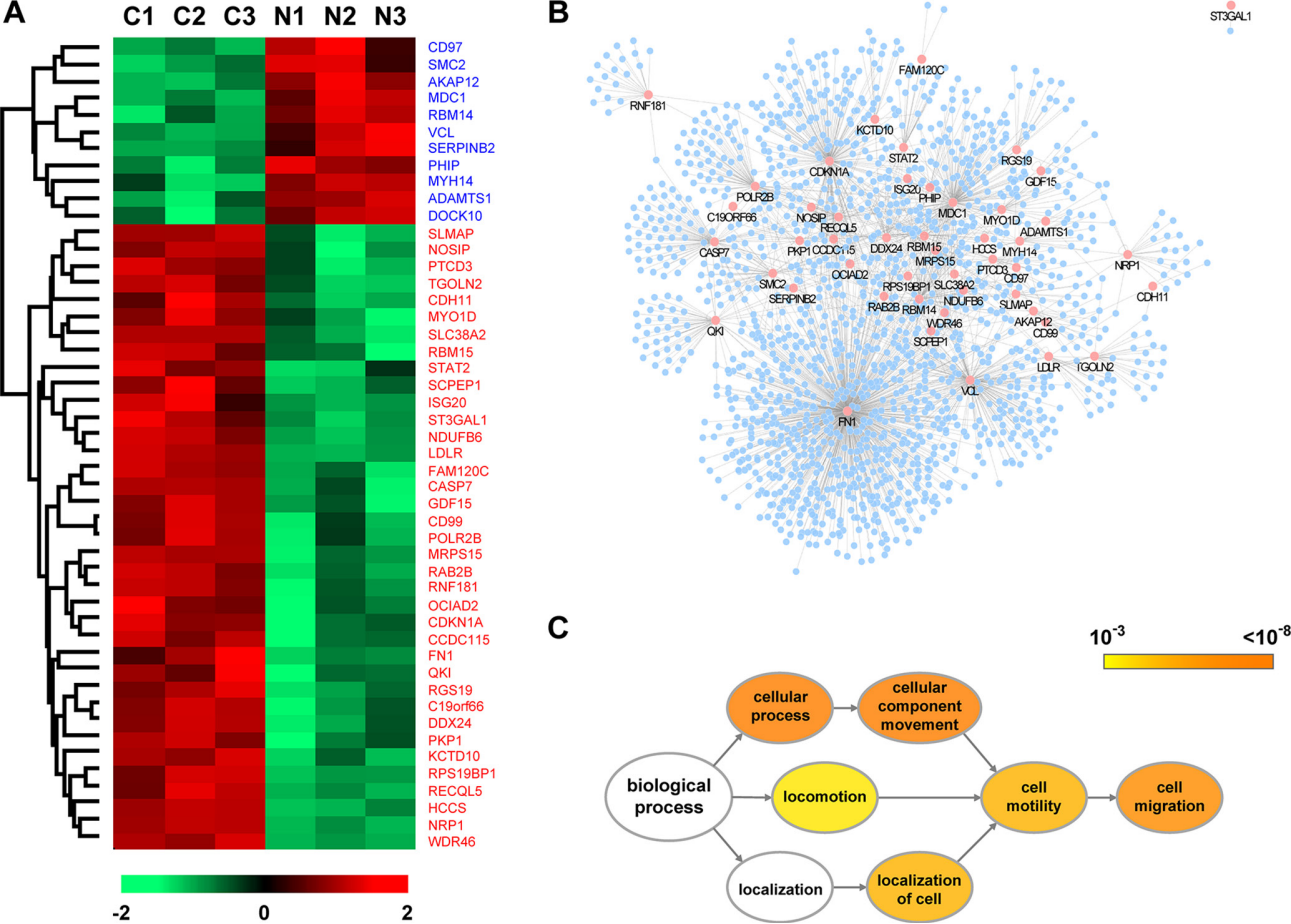
Identification of the key proteins contributing to the effect of NDGA on cell migration (**A**) Heat map of the significantly different proteins (SDPs) identified in proteomic assay with *p* < 0.01. Lane C1-C3 show the three replications of control set and lane N1-N3 present NDGA treated set. The color of cells represents expression level ranging from low (green) to high (red). Expression details of all SDPs could be found in Supplementary Table S1. (**B**) Protein-protein interaction (PPI) network derived from the SDPs mediated by NDGA. The PPI network is constituted of 1630 nodes and 2098 edges with pink nodes presenting SDPs. (**C**) Gene Ontology (GO) analysis was conducted upon the PPI network to enrich the nodes by their “biological processes” annotations. Diagram shows the structure of cell motility relevant GO terms and their root terms. The color of GO terms presents *p-value* and the size presents the number of enriched proteins.

To understand how NDGA exerts the inhibitory effect on cell migration, we employed a LC-MS/MS based quantitative proteomic assay to explore the proteins expression profile modulated by NDGA. In the control and NDGA treated groups, 3636 proteins were identified totally whit expression abundance quantified (Supplementary Table S1). Out of these proteins, 48 were significantly different proteins (SDPs) (*p* < 0.01) regulated by NDGA, among which 11 were up-regulated and 37 were down-regulated (Figure 2A). In order to identify the key proteins contributing to the suppression on cell migration, we constructed a protein-protein interaction (PPI) network basing on these 48 SDPs, which contains 1630 nodes and 2098 interactions (Figure 2B and Supplementary Material S1). Subsequently, Gene Ontology (GO) analysis was introduced to categorize all the proteins within the PPI network by the “biological process” they involved. The derived GO network contains 471 “biological process” GO terms (*p* < 0.001) (Supplementary Material S2 and Supplementary Table S2), among which we placed our focus on cell motility relevant GO terms “cell migration”, “cell motility” and their mother terms (Figure 2C). Intriguingly, we found two SDPs, NRP1 and FN, appear in all the cell motility relevant GO terms (Table 1). NRP1 and FN are down-regulated by NDGA according to the proteomics and they are the only two SDPs enriched into the child terms of “cell motility” and “cell migration”, implying that they play critical roles in the suppression of NDGA on cell migration.

**Table 1:** Details of cell motility relevant GO terms

GO-ID	Description	*P*-vaule	Cluster freq	Seed freq	SDPs
9987	Cellular process	1.18E-98	1318/1494	28/48	RAB2B,CD97,RBM14,RGS19,DDX24,SMC2,KCTD10,QKI,CDKN1A,ISG20,PKP1,SCPEP1,VCL,CD99,NDUFB6,GDF15,CDH11,CASP7,MRPS15,NRP1,PHIP,SLMAP,RECQL5,AKAP12,MDC1,LDLR,POLR2B,FN
6928	Cellular component movement	4.44E-12	100/1494	4/48	NRP1,FN,VCL,CD97
51674	Localization of cell	5.92E-08	65/1494	2/48	NRP1,FN
40011	Locomotion	8.16E-06	76/1494	2/48	NRP1,FN
48870	Cell motility	5.92E-08	65/1494	2/48	NRP1,FN
16477	Cell migration	1.53E-09	64/1494	2/48	NRP1,FN

### NDGA inhibits cell migration by suppressing the expression of NRP1

**Figure 3:**
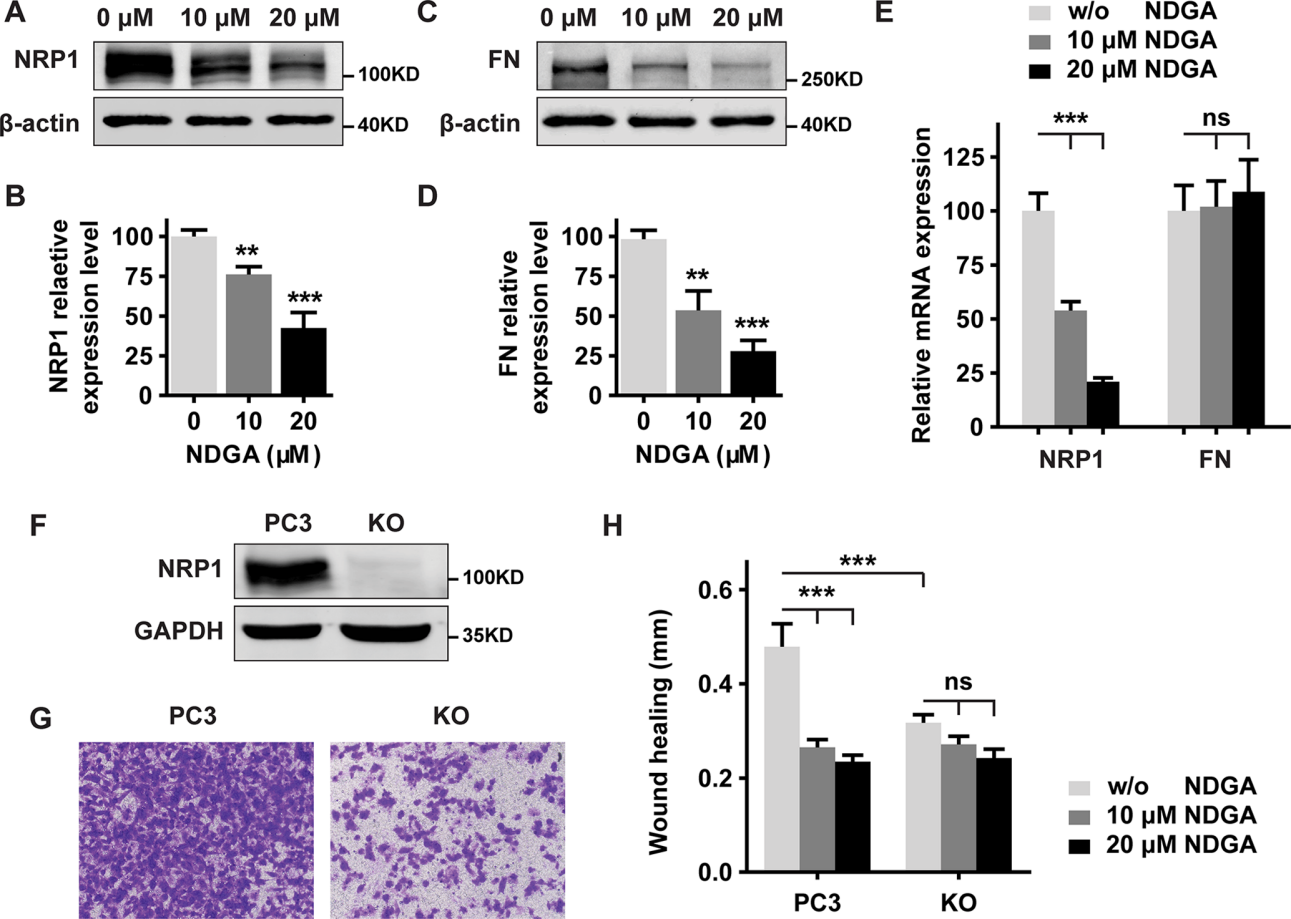
NDGA inhibits cell migration by suppressing NRP1 expression (**A**–**D**) Western blot analysis of PC3 cells proteins expression of NRP1 (A, B) and FN (**C**, **D**) with β-actin as the loading control. Statistical quantification of protein expression is shown as mean ± S.E (*n* = 3). **p < 0.01, ***p < 0.001. (**E**) mRNA expression levels of NRP1 and FN in PC3 cells measured by real time PCR. Data show mean ± S.E (*n* = 3). ***p < 0.001. (**F**) Verification of knockout efficiency in NRP1 knockout (KO) cell line generated from PC3 cells. (**G**) Cell motility of PC3 and KO cells evaluated by transwell assay. (**H**) Wound healing assay of PC3 and KO cells treated with NDGA for 24 hours. Quantification shows the absolute distance of migration presented as mean ± S.E (*n* = 3). ***p < 0.001.

To test the roles of NRP1 and FN in NDGA induced cell motility compromise, we firstly verified the change of their expressions. We found that NDGA treatment suppresses the protein expressions of both NRP1 and FN, which is consistent with our results in proteomic study (Figure 3A–3D). When examining the mRNA change, only the transcription of NRP1, but not FN, could be suppressed by NDGA (Figure 3E). Considering that NRP1 is reported to regulate the activities of integrins and to modulate FN fibril assembly [15], we hypothesized that NRP1 plays a more important role than FN in the function of NDGA. To prove our hypothesis, we knocked out NRP1 gene in PC3 cells using CRISPR-Cas9 genome editing system (Figure 3F, Supplementary Figure S1). As expected, KO (NRP1 knock out PC3 cell) cells show impaired cell motility compared with PC3 cells (Figure 3G), confirming the critical role of NRP1 in cell migration. Further, these two cell lines were treated with NDGA in parallel and we found that in contrast with PC3 cells, cell motility of KO cells does not further decrease after NDGA treatment (Figure 3H), strongly indicating that the effect of NDGA is largely mediated by NRP1 suppression.

### NDGA inhibits tumor cell-matrix adhesion and FAK activation

**Figure 4:**
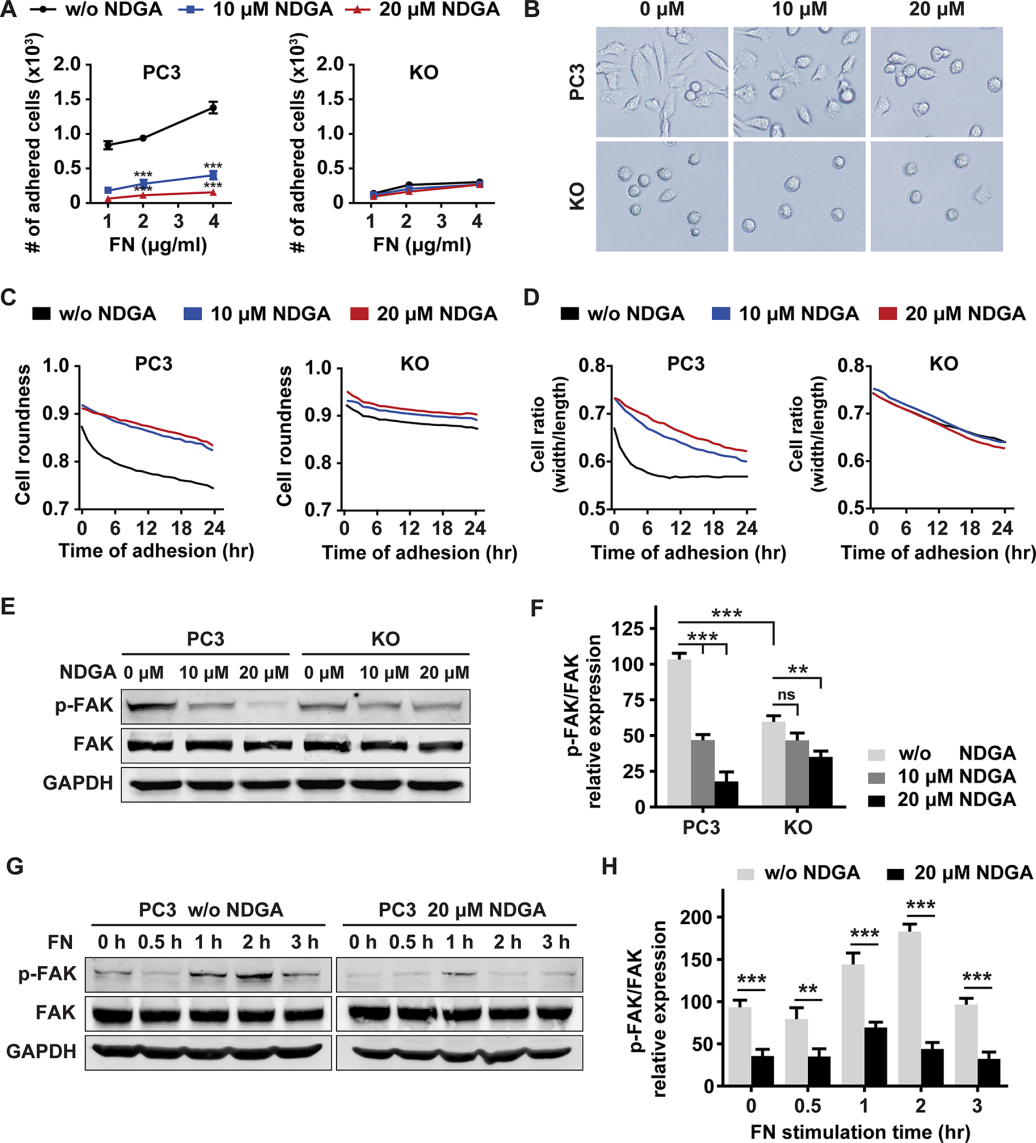
NDGA inhibits tumor cell-fibronectin adhesion and FAK activation (**A**) PC3 and KO cells were pretreated with 0, 10 or 20 μM NDGA for 24 hours and then seeded into FN-coated wells allowing for 30 minutes adhesion. Numbers of adhered cells of each group are counted. Data show mean ± S.E (*n* = 3). ***p < 0.001. (**B**) Pretreated cells were seeded into FN-coated wells and representative images of cell morphology after 24 hours adhesion were shown. (**C**, **D**) Morphological analysis of cells during adhesion progress. Cell adhesion to FN matrix was monitored in live imaging system for 24 hours. Cell roundness (C) and cell width/length ratio (D) of both PC3 and KO cells are shown. (**E**, **F**) Western blot analysis of p-FAK (Tyr379) and total FAK expression after NDGA treatment for 24 hours. All data are normalized to the control group of PC3 cells and results present mean ± S.E (*n* = 3). **p < 0.01, ***p < 0.001. (**G**, **H**) Western blot analysis of p-FAK (Tyr379) and total FAK expression levels under short term stimulation of FN in PC3 cells with or without 20 μM NDGA pretreatment. All data are normalized to the PC3 cells without FN stimulation. Data present mean ± S.E (*n* = 3). **p < 0.01, ***p < 0.001.

NRP1 is known to facilitate the interaction between tumor cells and microenvironment by affecting FN assembly [15, 16]. Therefore we further investigated whether NDGA impairs the adhesion of cells to ECM. PC3 and KO cells were treated with NDGA for 24 hours in parallel and we found that cancer cell-FN adhesion are impaired in NDGA treated PC3 cells and KO cells when compared to non-treated PC3 cells. However, NDGA treatment could not induce further impairment to cell adhesion in KO cells (Figure 4A). At the same time, cellular morphology during adhesion was monitored in live imaging system and cell roundness and cell width/length ratio were calculated to evaluate the adhesion progress. Similarly, we found that both NDGA treatment and NRP1 knockout notably affect cell morphology change during cell adhesion to ECM and delay the adhesion progress of PC3 cells (Figure 4B–4D). These results suggest that interactions between PC3 cells and FN are impaired by NDGA through NRP1 suppression.

When cell interacting with ECM, focal adhesion kinase (FAK) can be activated by integrin binding to FN and plays as a regulator of cell adhesion and migration [25]. Thus we tested the activation of FAK in PC3 and KO cells with or without NDGA treatment. In untreated cells, p-FAK (Tyr379) expression level of KO cells is lower than that of PC3 cells; when treated with NDGA, p-FAK expression level of PC3 cells decreases significantly but does not further decline in KO cells (Figure 4E, 4F). In addition to the long term FAK activation, we also tested the short term response of FAK activation to FN stimulation. Results show that activation of FAK reached the peak at 2 h and 1 h in control and NDGA pre-treated PC3 cells respectively after FN stimulation. However, the peak level of FAK activation in NDGA pre-treated cells is much lower than that in untreated control cells (Figure 4G, 4H). Taken together, our results show that NDGA inhibits cancer cell migration by blocking cell-ECM interaction and suppressing FAK activation, both of which are resulted from NRP1 suppression by NDGA.

### NDGA suppresses NRP1 expression and functionalities of endothelial cells and fibroblasts

**Figure 5:**
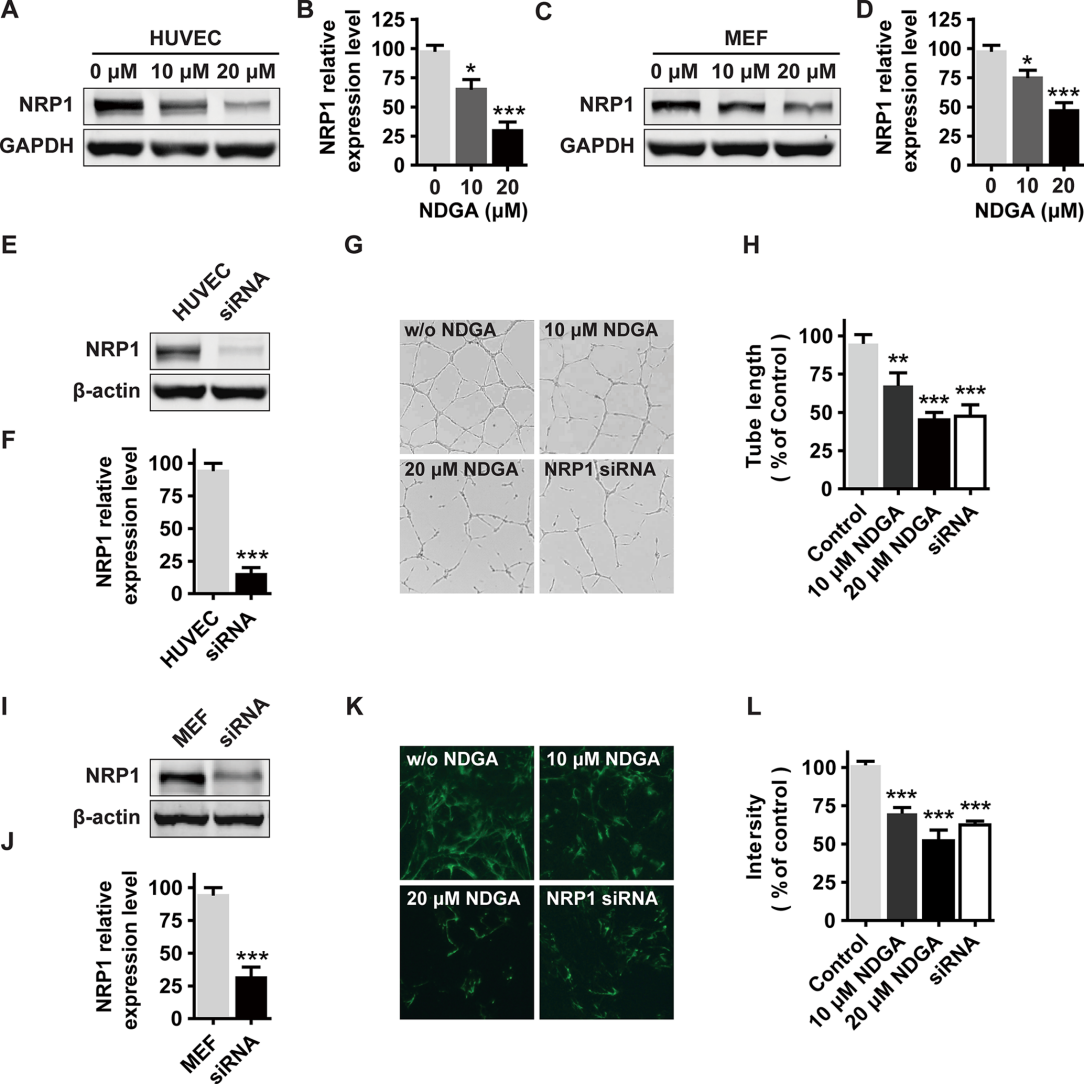
The effects of NDGA on endothelial cells and fibroblasts (**A**–**D**) NRP1 expression in HUVECs (A, B) and MEFs (C, D) under NDGA treatments. Data show mean ± S.E (*n* = 3). *p < 0.05, ***p < 0.001. (**E**, **F**) Verification of knockdown efficiency of NRP1 in HUVECs. Data show mean ± S.E (*n* = 3). ***p < 0.001. (**G**, **H**) HUVECs were seeded onto matrigel and treated with different concentrations (0, 10, 20 μM) of NDGA or transfected with siNRP1. Tube formation was assessed after 12 hours and representative images of the capillary tube like structures are shown. Data show mean ± S.E (*n* = 3). **p < 0.01, ***p < 0.001. (**I**, **J**) Verification of knockdown efficiency of NRP1 in MEFs. Data show mean ± S.E (*n* = 3). ***p < 0.001. (**K**, **L**) Immunofluorescence staining of FN fibril in MEFs that were treated with NDGA or transfected with siNRP1. Representative images are shown. Data show mean ± S.E (*n* = 3). ***p < 0.001.

Tumor progression involves not only tumor cells but also neighboring cells and ECM. Similarly, we found that NRP1 also functions in other types of cell and facilitates metastasis. As a co-receptor of VEGF, NRP1 is highly expressed in vascular endothelial cells and is critical in the regulation of tumor angiogenesis [26]. Besides, recent studies suggested that NRP1 also affects tumor microenvironment by facilitating integrin functions in fibroblasts [15]. Based on these information, we tested whether NDGA suppresses NRP1 and affects the functions of these two types of cell. The results show that NDGA treatment effectively suppresses NRP1 expression in both human umbilical vein endothelial cells (HUVECs) and mouse embryonic fibroblasts (MEFs) (Figure 5A–5D). Further, we tested the functionalities of HUVECs and MEFs by measuring their capabilities of tube formation and FN fibril assembly respectively. Results show that knocking down of NRP1 significantly attenuates tube formation activity of HUVECs (Figure 5E–5H) and polymerization of FN fibrils by MEFs (Figure 5I–5L), and NDGA treatment shows similar effects in both cell types. These results suggest that NDGA may suppress angiogenesis and ECM formation during metastasis progression and thus exert a comprehensive effects on tumor metastasis by affecting not only tumor cells but also the microenvironment.

### NDGA inhibits tumor growth and metastasis *in vivo*

**Figure 6:**
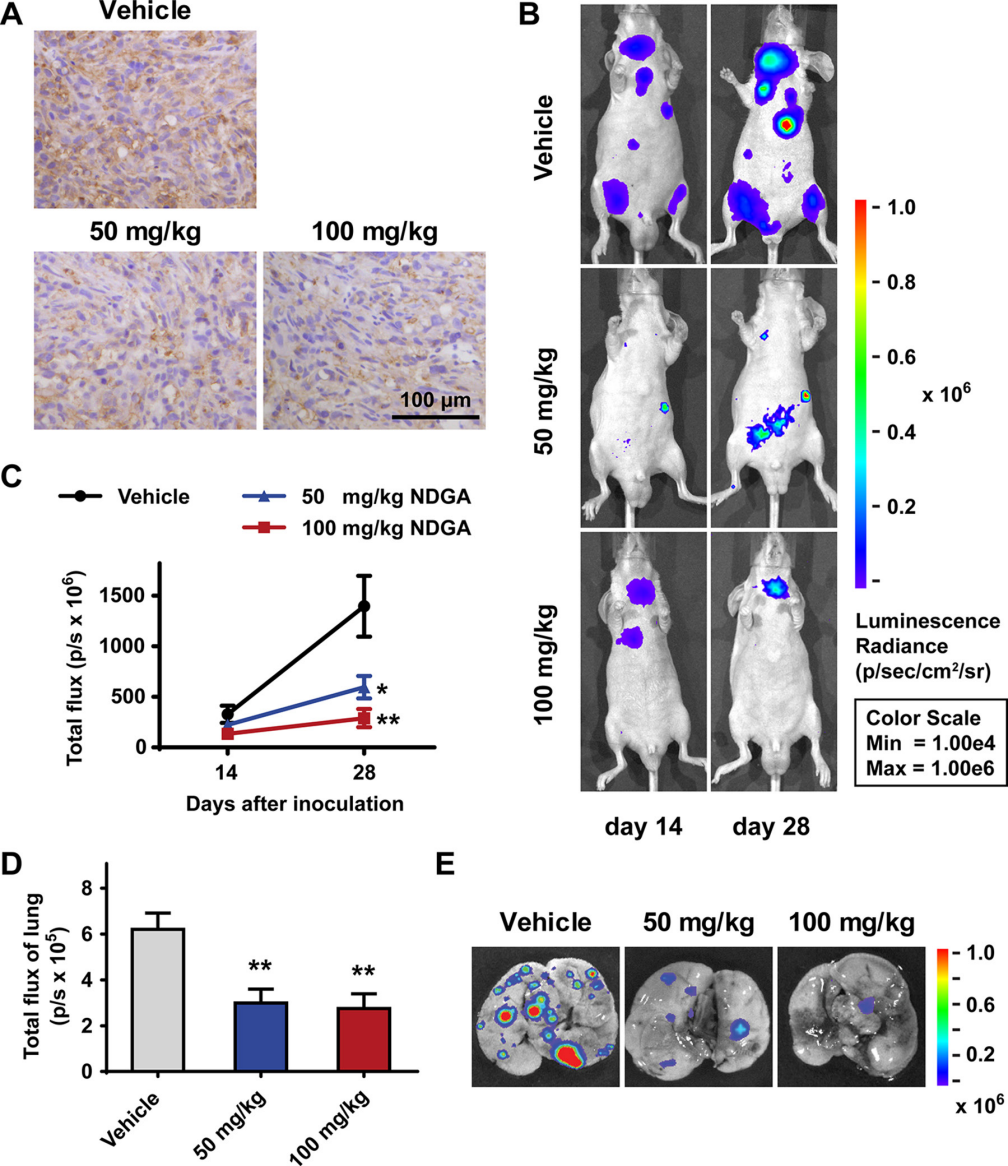
NDGA inhibits PC3 xenografts growth and metastasis (**A**) Immunohistochemistry staining of NRP1 in the tumor tissues of subcutaneous xenograft model. Scale bar: 100 μm. (**B**) Representative bioluminescence images showing the metastatic sites in intravenous injection model. Mice of three groups (vehicle, 50 mg/kg NDGA, 100 mg/kg NDGA) were monitored after 14 days and 28 days drug treatments. (**C**) Quantification of bioluminescence signal within the region of interest at day 14 and day 28. Data show mean ± S.E (*n* = 5). *p < 0.05, **p < 0.01. (**D**) Quantification of bioluminescence signal of lungs at day 28. Data show mean ± S.E (*n* = 5). **p < 0.01. (**E**) Representative bioluminescence images of lungs with metastatic sites.

At last, we tested the anti-metastasis activity of NDGA *in vivo* employing tumor xenograft models on nude mice. Firstly we inoculated tumor cells subcutaneously into the flank of nude mice to test whether NDGA administration affects NRP1 expression *in vivo*. Results show that NRP1 expression level in tumor tissues are lower in NDGA treated groups (Figure 6A), consistent with our *in vitro* results. Besides, we also observed compromised tumor growth in NDGA treated groups (Supplementary Figure S2). To evaluate tumor metastasis, luc-PC3 cells were injected into tail vein of the nude mice and tumor metastasis was evaluated by bioluminescence. Results show that NDGA treatment significantly suppresses tumor metastasis in whole body range (Figure 6B, 6C). Further, after 4 weeks the mice were sacrificed and metastatic nodules on lungs were observed by bioluminescence. Again, we found less metastatic sites in NDGA treated mice than control (Figure 6D, 6E). By these results, we can further confirm the inhibitory effect of NDGA on tumor metastasis.

## DISCUSSION

Metastasis is the master hallmark of malignant tumors and is largely incurable leading to more than 90% mortality for cancer patients [2]. For decades of study, it has been recognized that both the natures of tumor cell and its crosstalk with surrounding environment conduct the metastatic cascade; and thus numerous relevant pathways and proteins have been identified as therapeutic targets [27]. However, there are still many problems to be solved to make a full translation of these basic researches to clinical applications and more efforts to be made to develop novel therapeutic strategies for metastatic diseases [28, 29].

In our study, we demonstrated that the NDGA impairs tumor cell motility through suppressing NRP1, revealing a novel functional target of NDGA. According to previous studies, NDGA shows widespread therapeutic potentials in diverse diseases and disorders by targeting multiple molecules and pathways, which is one of the common characteristics of natural products. In cancer models, NDGA has been demonstrated to show antineoplastic effects by inhibiting cell proliferation and inducing apoptosis through different mechanisms [20, 22]. Specifically, NDGA has been identified as inhibitor of LOX, IGF-1R and HER2, influencing the related signaling pathways and suppressing tumorigenesis and growth [7, 8]. Despite of these discoveries, the majority of current understandings of NDGA functions in cancer biology are focused on its anti-proliferation activity, while very few studies involve its effects on cell migration and tumor metastasis [30]. In our study, we detected that NDGA significantly suppresses PC3 cell migration at the concentrations that have no influence on cell proliferation. Some of the known functional targets of NDGA, such as LOX and IGF-1R, are also known to be involved in regulation of cell motility [31, 32]. However, some other inhibitors of these targets do not affect cancer cell mobility in our model, strongly suggesting that NDGA suppresses cell mobility through a novel pathway.

By proteomic study and network analysis, we recognized NRP1 as the potential target that contributes to the anti-migration activity of NDGA. Actually, according to the GO analysis, both FN and NRP1, suppressed by NDGA treatment, are capable to be the key proteins regulating the action of NDGA. NRP1 is known to regulate cell adhesion by interacting with integrins and promoting their functions [33, 34]. Fibronectin is an essential component of extracellular matrix, whose assemble and degradation are tightly regulated by integrins [35, 36]. It is reported that NRP1 promotes integrin α5β1-dependent fibronectin fibrils assembly, matrix stiffness and tumor growth [15, 37]. Meanwhile in our subsequent experiments we detected that although the protein expressions of both FN and NRP1 are decreased by NDGA, only the NRP1 transcription is impaired. So basing on these knowledge, we hypothesized that NRP1 suppression is critical for the inhibition of NDGA on cell motility and is also responsible for the FN inhibition. Moreover, in the NRP1 knockout cells, the protein expression of FN is low than that of wild type PC3 cells. Then we demonstrated that the motility of NRP1 knockout cells is impaired seriously and meanwhile the influence of NDGA almost disappeared. These results strongly support our prediction of network analysis and confirm NRP1 as a novel therapeutic target for NDGA.

Nevertheless, NRP1 was firstly found as an adhesion molecular [38] and recent studies have delineated the details of NRP1 activity in cell adhesion and relevant biological processes. It is reported that NRP1 promotes integrin α5β1-mediated endothelial cells adhesion to fibronectin by facilitating the Rab5/GIPC1/Myo6-dependent internalization of active integrin α5β1 [16]. Another group reported that NRP1 augments the FN fibril assembly activity of integrin α5β1 by interacting with GIPC1 and c-Abl using its SEA motif and activates tumor microenvironment [15].These results provided evidence that NRP1 regulates cell adhesion dependent on integrin α5β1 function. It is also reported that inhibiting NRP1 using monoclonal antibodies could inhibit the formation of integrin α5β1-NRP1 complex, the activation of FAK pathway and the adhesion of tumor cells to fibronectin [39, 40]. FAK, in control of cell adhesion and cell motility, is the central downstream signaling pathway of integrin and can be activated by the binding of integrin and FN [25]. In our study we detected that both the adhesion ability and the FAK activation level are impaired in the KO cells and that NDGA treatment alleviates the adhesion ability and the activation of FAK in PC3 cells, but have no influence on KO cells. These results imply that NDGA inhibits cell adhesion and FAK activation by inhibiting NRP1. When under the short term stimulation of FN, the FAK activation in NDGA treated cells is much milder than that in control cells, indicating that the function of integrins is impaired under the NDGA treatment. Based on the studies mentioned above, our results strongly suggest that NDGA suppresses cell migration through a NRP1-integin-FAK signaling cascade.

Metastasis is a complex biological process involving the modulations from many aspects [2]. In addition to the aggressive nature of tumor cells, the interactions between tumor cells and other types of somatic cells also play important roles [41]. In a recent study, NRP1 is reported to promote FN assembly in fibroblast cells, resulting in a metastasis-promoting microenvironment [15]. On the other hand, NRP1 also facilitates angiogenesis, another essential event in the progression of metastasis, as a co-receptor of VEGF [26]. In our study, NDGA shows similar function on NRP1 suppression in both fibroblast and vascular endothelial cells. Moreover, NDGA strongly attenuates the FN fibril assembly and angiogenesis in two cell types respectively. These findings suggest that NDGA has diverse effects on multiple cell types and may play a comprehensive role in suppressing tumor metastasis.

Consistent with the findings in cancer biology research, NRP1 is also tightly linked with tumor progression in clinic. NRP1 is frequently overexpressed in several tumor types including carcinomas (e.g., prostate, breast, colon, kidney, pancreas), melanoma, glioblastoma and others, generally correlating with aggressive clinical tumor behavior and poor prognosis [11, 26, 42–44]. In some cancer types, the frequency of NRP1 positivity is much higher in metastatic tumor than that in primary tumor [45]. In *in vivo* cancer models, NRP1 has also been demonstrated to be critical in promoting tumor metastasis [46, 47]. In our work we also tested the function of NDGA in xenograft mice models. Using subcutaneous xenograft model we confirmed that NDGA suppresses NRP1 expression in tumor tissue. And in tail vein injection model we observed attenuated tumor metastasis after NDGA treatment.

NRP1 has been demonstrated to be a potential target in cancer therapies. Small molecule inhibitor, specific antibody or short peptide targeting NRP1 have been shown to suppress tumor growth and metastasis [17–19]. Furthermore, a human anti-NRP1 monoclonal antibody, MNRP1685A, has finished its phase I clinical trials in advanced solid tumors [48]. In our study, we demonstrated that NDGA treatment can strongly block the expression and consequently the function of NRP1. Besides prostate cancer PC3 cell model, we also detected similar pharmacological effects of NDGA on NRP1 expression and cell migration in breast cancer MDA-MB-231 cells, which also show high level of NRP1 expression and cell migration. Taken together, our results indicate the potential application value of NDGA in cancer therapies targeting NRP1 in selected tumor types with NRP1 overexpression.

## MATERIALS AND METHODS

### Regents and antibodies

Sources of chemicals are as follows: NDGA, caffeic acid (Sigma, USA); AG538 (Cayman, USA); picropodophyllin (PPP, Selleck, USA), fibronectin protein (FN, Santa Cruz, USA). Antibodies against NRP1 (diluted 1:1000), FN (diluted1:1000), β-actin (diluted 1:10000) and GAPDH (diluted 1:1000) were purchased from Abcam, USA. Antibodies against total FAK (diluted 1:1000), phospho-FAK (Tyr397) (diluted 1:1000), DyLight 800-conjugated secondary antibodies (diluted 1:10000), DyLight 488-conjugated secondary antibodies (diluted 1:1000) and HRP-conjugated secondary antibodies (diluted 1:1000) were purchased from Cell Signaling Technology, USA.

### Cell lines and cell culture

PC3 cell was purchased from Chinese Academy Science Cell Bank (Shanghai, China). Luc-PC3 cell was a gift from Dr. Yuqian Zhang (Cancer Hospital Chinese Academy of Medical Science, Beijing, China). The two cell lines above were maintained in high-glucose Dulbecco's Modified Eagle's Medium/ Nutrient Mixture F-12 (DMEM/F-12, HyClone, USA) supplemented with 10% fetal bovine serum (FBS, Gibco, USA), 100 U/ml penicillin and 100 μg/ml streptomycin. Mouse embryonic fibroblasts (MEFs) were isolated from 13.5 days mouse embryo and cultured according to the protocol as previously described [49]. Human umbilical vein endothelial cells (HUVECs) were purchased from Lonza (Basel, Switzerland) and cultured in Endothelial Growth Medium (EGM-2, Lonza). All cells were maintained in 5% CO_2_ incubator at 37°C.

### Generation of NRP1 knockout cell line using CRISPR-Cas9 system

SgRNA sequences (top: 5′-caccgctgtcctccaaatcga agtg-3′, bottom: 5′-aaaccacttcgatttggaggacagc-3′) targeting exon 2 of NRP1 gene were designed (http://tools.genomeengineering.org) and cloned into pSpCas9(BB)-2A-GFP plasmid (Addgene plasmid #48138) following the procedure previously described [50]. PC3 cells were transfected with the recombined sgRNA-pSpCas9(BB)-2A-GFP plasmid and GFP-positive cells were deposited into 96-well plate by FACSAria II cell sorter (BD Biosciences, USA) for expansion. Knockout efficiency of monoclonal cell lines are detected by Western blot.

### siRNAs and transfection

Cells at 60–80% confluence were transiently transfected with siRNA using Lipofectamine^®^ RNAiMAX (Invitrogen, USA). The siRNA targeting human NRP1 and mouse NRP1 were purchased from Santa Cruz, USA.

### Cell proliferation assay

Cells were seeded into 96-well plate and cultured. After adhesion, cells were treated with indicated drugs for 24 hours. Cell proliferation was determined using CellTiter 96^®^ AQueous One Solution Cell Proliferation Assay (MTS) (Promega, USA) following the product instruction.

### Wound healing assay

Cells were firstly cultured into confluent monolayer. Then a scratch was made using P200 pipette tip, following which cells were treated with indicated drugs. The width of the wound was monitored at 0 hour and 24 hours after scratching using microscope (Nikon, UK) and the closure area was quantitated using ImageJ software (NIH, https://imagej.nih.gov/ij/) to assess the capacity of cell migration.

### Transwell assay

Cells were seeded into the upper chamber of a Transwell (Corning, USA) insert with indicated treatments and culture medium was added into the lower chamber. After indicated time, cells in the upper face of the chamber were removed by cotton swabs and migrated cells on the lower surface were stained with crystal violet and counted to assess cell motility.

### Cell adhesion assay

Cells pretreated with indicated drugs for 24 hours were suspended and seeded into 96-well plate which was pre-coated with human FN. After 30 min incubation, culture medium was removed and cells adhered on plate surface were stained with crystal violet, photographed and then counted using ImageJ software. To further evaluate the adhesion progress, after seeded into plate, cells were cultured in live imaging system (Operetta High Content Imaging System, Perkin-Elmer, Germany) and cellular morphology change over 24 hours were analyzed. Cell roundness and cell width/length ratio were calculated to assess cellular morphological feature indicating the progress of adhesion.

### Tube formation assay

HUVECs were seeded in to 24-well plate pre-coated with growth factor-reduced matrigel (BD Biosciences, USA). Cells were incubated with indicated treatments for 8 hours, following which the capillary tube like structures formed by HUVECs were photographed under microscope.

### LC-MS/MS based proteomic assay

LC-MS/MS based proteomic analysis of cell content was conducted as described previously [51]. Briefly, proteins in cell lysate were digested into small peptides using trypsin and the peptides were subsequently identified by LC-MS/MS system (LTQ-Orbitrap Velos mass spectrometer, Thermo Fisher Scientific, Germany) and recognized in Uniprot human protein database (http://www.uniprot.org/). The relative abundance of identified proteins was calculated using MaxQuant (http://www.maxquant.org/) and student's *t* test was conducted to analyze significantly different proteins (SDPs).

### Networks construction and analysis

To determine the key proteins that contribute to the function of NDGA on prostate cancer cell migration, protein-protein interaction (PPI) network was constructed basing on the SDPs identified in the proteomic assay and visualized using Cytoscape platform as previously described [51]. Subsequently, Gene Ontology (GO) analysis was conducted to enrich the proteins within the PPI network for biological processes.

### Western blot

The Western blot procedure was carried out as previously described [52]. Briefly, cells were lysed in RIPA buffer containing PMSF and protease inhibitor mixture. Then 60 μg of protein lysate from each sample was loaded to SDS-PAGE gel and transferred to PVDF membrane for detection of specific proteins. The membrane was incubated in primary antibody at 4°C overnight and then in Dylight-800 conjugated secondary antibody at room temperature for 1 hour. The signals were finally detected using Odyssey infrared imaging system (LI-COR Biosciences, USA) and quantified using ImageJ software.

### Real-time PCR

Total RNA was extracted using TRIzol reagent (Invirtogen, USA) according to the instruction from the manufacturer, after which cDNA was synthesized using RevertAid First Stand cDNA Synthesis Kit (Fermentas, USA). Real-time PCR was performed using SYBR Green RealTime PCR Master Mix (Promega, USA) in Mx3005P system (Agilent Technologies, USA). The sequences of primers were as follows: NRP1, forward 5′-ATCACGTGCAGCTCAAGTGG-3′ and reverse 5′-TCA TGCAGTGGGCAGAGTTC-3′; FN, forward 5′-GAGA ATAAGCTGTACCATCGCAA-3′ and reverse 5′-CGAC CACATAGGAAGTCCCAG-3′; GAPDH, forward 5′-GGA GCGAGATCCCTCCAAAAT-3′ and reverse 5′-GGCTG TTGTCATACTTCTCATGG-3′.

### Immunohistochemistry staining

Immunohistochemistry staining was performed as previously described [53]. Briefly, sections (4 μm) from paraffin-embedded tissue were incubated in primary antibody at 4°C overnight and then in HRP-conjugated secondary antibody for 1 hour. Then sections were incubated with diaminobenzidine and further stained with hematoxylin to show nuclei.

### Immunofluorescence staining

Immunofluorescence staining was performed as previously described [15]. Briefly, cells were seeded onto coverslips and treated with indicated drugs for 24 hours. Then cells were fixed and incubated in primary antibody at 4°C overnight and then in DyLight 488-conjugated secondary antibody. Images were captured under fluorescence microscope (Olympus, Japan).

### Animal models

All procedures involving animals were conducted according to the European community guideline for the use of experimental animals and approved by the Peking University Committee on Animal Care and Use. All efforts were made to minimize animal suffering. Four-week-old male immunodeficiency mice were subcutaneously or intravenously injected with 5 × 10^6^ PC3 cells or luc-PC3 cells respectively and then randomized into three groups orally administered with indicated drugs or vehicle (0.5% carboxymethylcellulose sodium). In subcutaneous xenograft model, tumor growth was measured as previously described [53]. In intravenous injection model, to monitor whole body metastasis, mice were anaesthetized with 3% isoflurance and intraperitoneally injected with 150 mg/kg D-luciferin. The bioluminescent images of mice were captured by IVIS^®^ Spectrum imaging system (PerkinElmer, USA) and the bioluminescence intensity of region of interest (ROI) were calculated within the system.

### Data statistics

All experiments were performed independently at least three times. Data were shown as mean ± standard error (S.E). Student's *t-test* and one-way analysis of variance (ANOVA) were performed to analyze the statistical significance with *p < 0.05, **p < 0.01 or ***p < 0.001.

## SUPPLEMENTARY MATERIALS FIGURES AND TABLES







## Abbreviations

NDGA: nordihydroguaiaretic acid
NRP1: neuropilin 1
PPI network: protein-protein interaction network
GO: gene ontology
ECM: extracellular matrix
LOX: lipoxygenase
IGF-1R: insulin-like growth factor 1 receptor
FN: fibronectin
FAK: focal adhesion kinase
