# Supplementary material for: Nordihydroguaiaretic acid impairs prostate cancer cell migration and tumor metastasis by suppressing neuropilin 1
Source: Oncotarget. 2016 Nov 15;7(52):86225–38. doi: 10.18632/oncotarget.13368 (PMC5349909; doi:10.18632/oncotarget.13368)
Supplement: Supplementary file 1 [file oncotarget-07-86225-s001.pdf]

Nordihydroguaiaretic acid impairs prostate cancer cell migration and tumor metastasis by suppressing neuropilin 1

Supplementary Materials

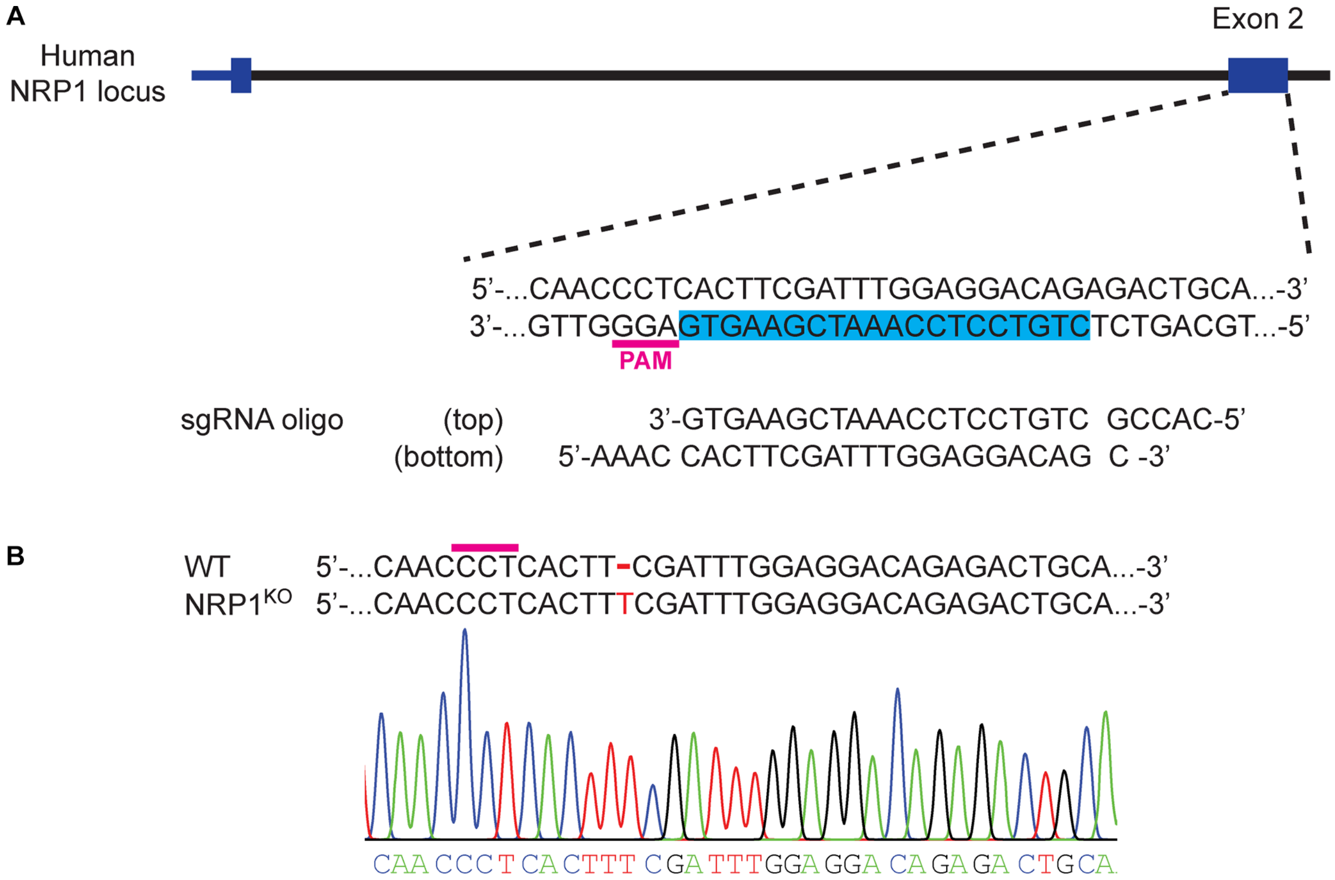

Supplementary Figure S1: CRISPR-Cas9 meditated NRP1 knockout. (**A**) The exon 2 of human NRP1 gene was targeted and the sequences of sgRNAs (top and bottom) are shown. (**B**) The DNA base pairs adjacent to the supposed double strands break site was sequenced. A 1bp insertion was induced in exon 2 leading to frameshift of NRP1 coding sequence.

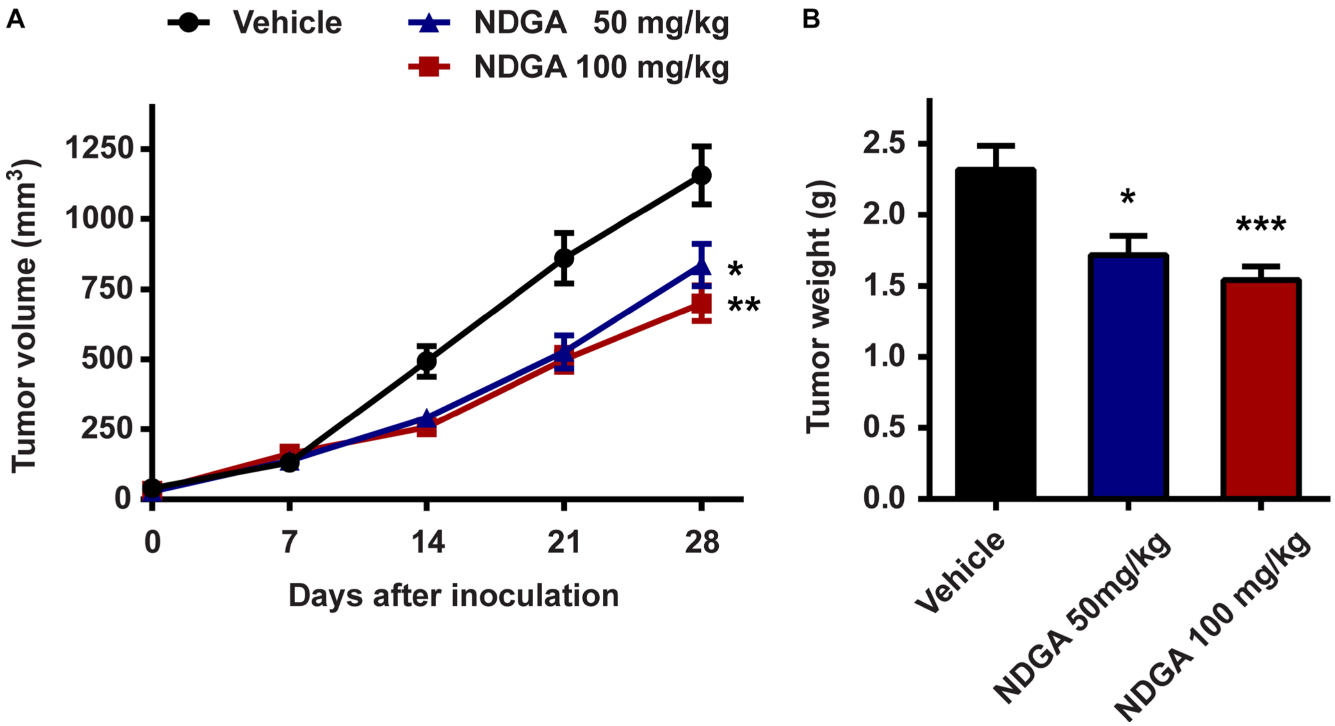

Supplementary Figure S2: The effects of NDGA on tumor growth in subcutaneous xenograft model. (**A**) Nude mice were subcutaneously injected with PC3 cells and orally administrated with vehicle, 50 mg/kg NDGA or 100 mg/kg NDGA. Tumor volume was measured at indicated times and calculated as $V = (\text{width}^2 \times \text{length})/2$. Data show mean ± S.E ($n = 5$). $*p < 0.05$, $**p < 0.01$. (**B**) Tumor weight was measured at the endpoint of experiment. Data show mean ± S.E ($n = 5$). $*p < 0.05$, $***p < 0.001$.

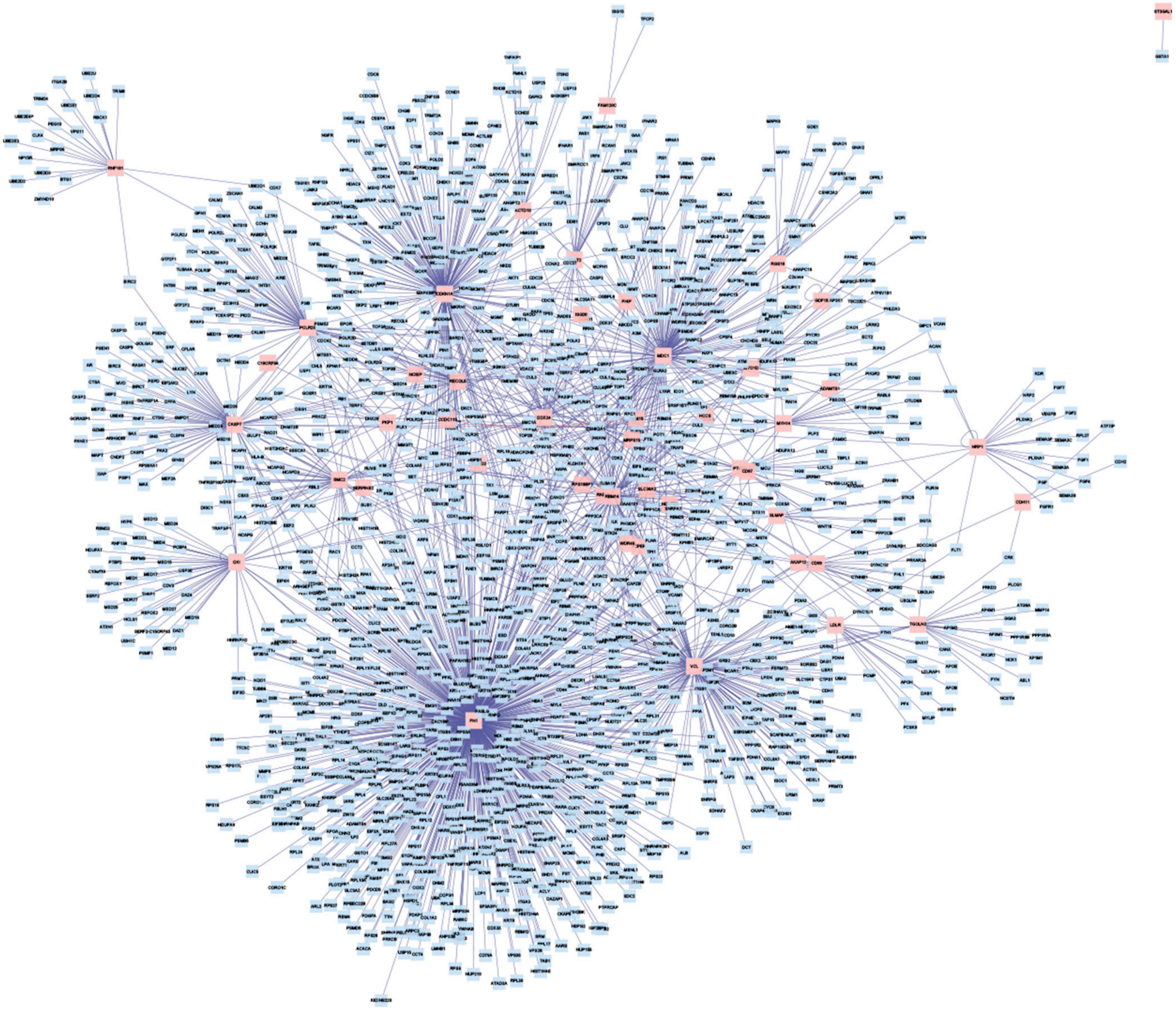

Supplementary Material S1: The details of protein-protein interaction network

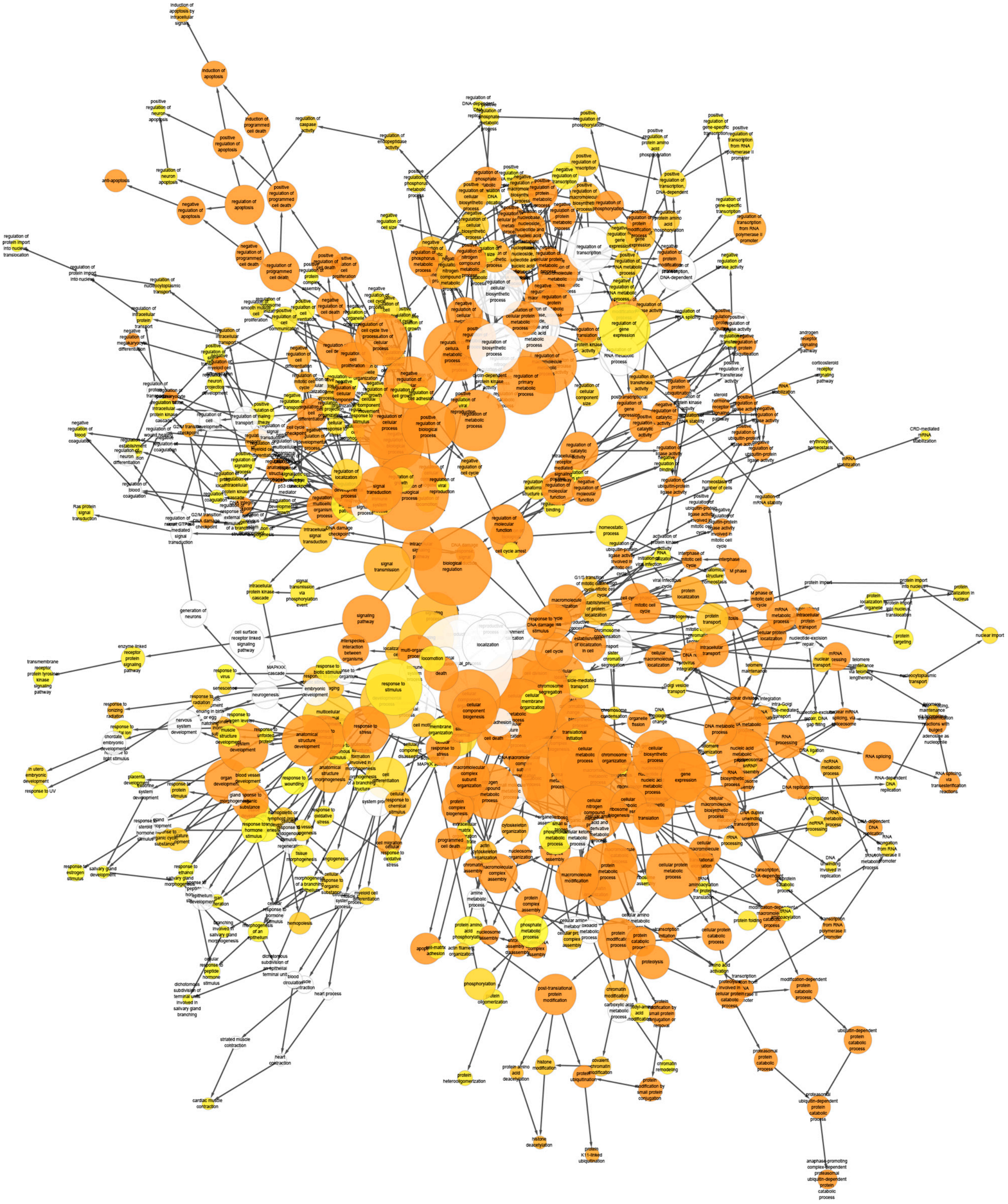

Supplementary Material S2: The details of Gene Ontology analysis

Supplementary Table S1: Result of proteomic assay. See Supplementary_Table_S1

Supplementary Table S2: Details of GO analysis. See Supplementary_Table_S2
